# A mouse model of neoadjuvant chemotherapy followed by interval cytoreductive surgery indicates impaired efficacy of perioperative cisplatin

**DOI:** 10.1186/s13048-021-00895-w

**Published:** 2021-11-16

**Authors:** Mitchell Clark, Alexandra Kollara, Theodore J. Brown, Taymaa May

**Affiliations:** 1 Division of Gynecologic Oncology, Princess Margaret Cancer Centre, 700 University Avenue, Room 6-811, Toronto, ON M5T 1Z5 Canada; 2 Department of Obstetrics and Gynaecology, University of Toronto, Toronto, ON Canada; 3 Present address: Division of Gynecologic Oncology, Department of Obstetrics, Gynecology and Reproductive Sciences, Yale School of Medicine, 333 Cedar Street, New Haven, CT USA; 4 Lunenfeld-Tanenbaum Research Institute, Mt. Sinai Hospital, Toronto, ON Canada

**Keywords:** Ovarian cancer, Neoadjuvant chemotherapy, Cisplatin, Cytoreductive surgery, Mouse, ID8 cells, Ovarian neoplasms, Neoadjuvant chemotherapy treatment, Surgical oncology, Animal model

## Abstract

**Background:**

Investigate the impact of interval cytoreductive surgery (ICS) on progression in an orthotopic mouse model of ovarian cancer and the impact of chemotherapy delivered at various timelines following surgery.

**Methods:**

Luciferase-expressing ID8 murine ovarian cancer cells were implanted intra-bursally and IP to C57BL/7 mice. Once disease was established by bioluminescence, 2 cycles of neoadjuvant cisplatin were administered, and animals received either ICS (removal of the injected bursa/primary tumor) or anesthesia alone. Postsurgical chemotherapy was administered on the same day as the intervention (ICS/anesthesia), or on day 7 or day 28 following the intervention. Progression was quantified serially with in vivo bioluminescence imaging. Volume of ascitic fluid volume collected at necropsy was measured.

**Results:**

Animals were matched for tumor burden at stratification. There was no accelerated growth of residual tumor after interval cytoreduction compared to controls. Animals who received chemotherapy on postoperative day (POD) 7 had better disease control compared to standard-of-care POD 28. Animals who underwent surgery had less ascites at necropsy compared to those who had anesthesia alone.

**Conclusions:**

In this animal model, surgical wounding with suboptimal cytoreduction after neoadjuvant chemotherapy did not cause accelerated expansion of residual disease. Surgical wounding appears to impair cisplatin activity when given at time of surgery.

## Background

Epithelial ovarian cancer continues to represent the most lethal gynecologic malignancy, with over 22,000 women diagnosed annually in the United States alone and approximately 14,000 succumbing to their disease [1]. Approximately 70% of women present with advanced-stage disease involving diffuse peritoneal spread [2]. Primary cytoreductive surgery followed by adjuvant platinum-based chemotherapy is generally the frontline standard of care [3]. While the survival benefits of surgery are well documented and the amount of residual disease at the completion of surgery is one of the strongest prognostic indicators for survival, optimal cytoreduction is not always achievable [4]. Removal of all visible tumor cells offers the best survival outcomes, whereas suboptimal cytoreduction, defined as residual disease at the conclusion of surgery of greater than 1 cm^3^, offers no improvement in overall survival [5, 6]. Thus, those patients who have presumed unresectable disease or who are judged to be poor surgical candidates are best offered neoadjuvant chemotherapy (NACT) followed by interval cytoreduction and post-operative adjuvant consolidation chemotherapy [7, 8].

Despite the well-established advantages of cytoreductive surgery, multiple studies demonstrate that surgical wounding can lead to the release of factors that promote proliferation and metastasis of residual tumor cells in various cancers [9–17]. This negative aspect may diminish the full potential benefit of surgical cytoreduction. In non-gynecologic malignancies, it has been shown that surgical removal of a primary tumor increases the growth of metastatic foci, and increases the proportion of quiescent malignant cells recruited into active replication [15, 18–20]. Several mechanisms have been identified that could explain the increased tumor burden [reviewed in [21]], including the release of tumor cells into the circulation at the time of surgery, impaired immune function secondary to a systemic inflammatory response, and potentiation of quiescent metastatic foci. In addition, evidence from animal models indicates that the primary tumor in various malignancies may exert an inhibitory effect on the growth of metastatic disease, through the regulation of angiogenesis [22, 23]. Surgical excision of the primary tumor removes this suppression, thereby enabling growth acceleration of metastatic lesions.

Our group has previously examined this concept in an ovarian cancer mouse model mimicking primary cytoreductive surgery [17]. Using an ID8 syngeneic model of ovarian cancer [24, 25] modified to mimic residual disease following optimal primary cytoreduction, we demonstrated that incisional wounding accelerated tumor growth and decreased perioperative cisplatin efficacy [17]. It is reasonable to speculate that impaired cisplatin efficacy might contribute to the clinical study findings of no clear benefit of intraoperative hyperthermic chemotherapy in ovarian cancer patients undergoing primary cytoreductive surgery [26]. In contrast, some benefit of intraoperative hyperthermic chemotherapy has been shown in patients undergoing interval cytoreductive surgery following NACT [27].

Despite the increased use of NACT in the treatment of advanced ovarian cancer [28], there are limited data on the interplay between exposure to chemotherapy and the response to surgical wounding at interval cytoreduction. Given the well-established myelosuppressive effects of platinum-based chemotherapy and the role of bone marrow-derived progenitor cells in the wound healing response, the impact of incisional wounding may be different in a NACT model of ovarian cancer, when combined with tumor cytoreduction. In this study, we evaluated the impact of interval cytoreductive surgery (ICS) in an immunocompetent orthotopic mouse model of advanced ovarian cancer using the syngeneic ID8 murine ovarian cancer cell line and examined the impact on perioperative cisplatin treatment.

## Results

**Fig. 1:**
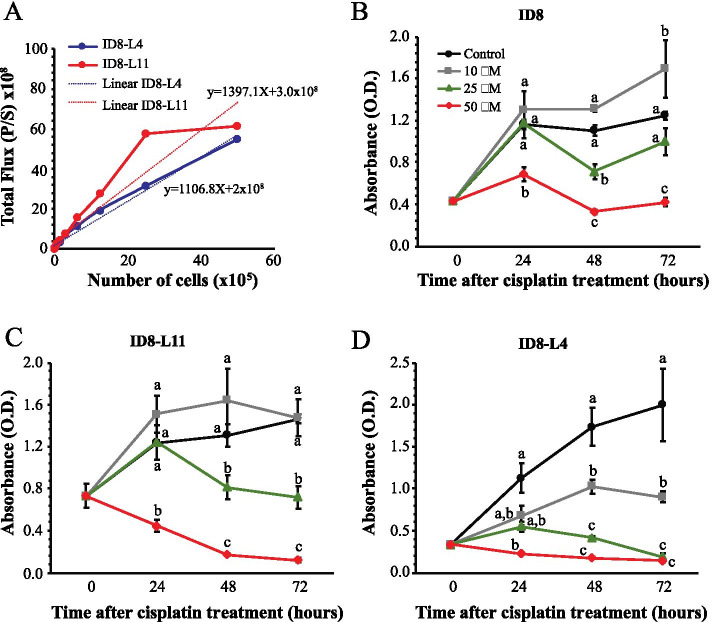
Comparison of ID8-L4 and ID8-L11 cells for linearity of bioluminescence output and cisplatin sensitivity *in vitro*. **A** Graphical representation of concentration-dependent bioluminescence imaging. Different concentrations of ID8-L11 and ID8-L4 cells were seeded into a 96-well plate and imaged on an IVIS spectrum *in vivo* imaging system. Measured values are shown with solid lines. Linear fitting of the data was performed and is represented by dashed lines. **B-D** ID8 (**B**), ID8-L11 (**C**) and ID8-L4 (**D**) cells were seeded in 96 well plates and 24 h later were treated with different concentrations of cisplatin (0, 10, 25 or 50 μM). Cell viability was determined by XTT dye reduction assays at the time points indicated. Data shown indicate the mean ± SEM of 4 independent replicates. Within each time point, groups with different letters are statistically different from one another as determined by ANOVA followed by Fisher’s LSD test (*p*<0.05)

Clonally selected ID8 cell sublines stably transfected with a luciferase expression construct driven by a constitutive CMV promoter were previously generated [17]. Luciferase and in vivo cell growth assays were performed to identify the most appropriate engineered ID8 subline exhibiting a linear bioluminescence response and maintained sensitivity to cisplatin for use in the *in vivo* study. Of these sublines, ID8-L11 and ID8-L4 cells were found to have the greatest overall levels of luciferase activity. Serially diluted concentrations of ID8-L11 and ID8-L4 cells seeded into a 96-well plate were imaged with the same system used to live image bioluminescent tumors. Of the two clonal sublines, ID8-L11 cells expressed greater activity; however, ID8-L4 cells exhibited a linear relationship over a greater range of cell concentrations (Fig. 1A). These same clones were compared for cisplatin sensitivity using an XTT dye-reduction assay. Both parental ID8 cells and ID8-L11 cells were growth inhibited by 25 or 50 μM cisplatin (Fig. 1B and C), whereas ID8-L4 cells exhibited a clear dose-dependent effect of cisplatin with decreased growth detected with as little as 10 μM cisplatin (Fig. 1D). Based on their apparent greater sensitivity to cisplatin and linearity of bioluminescence over a greater cell density range, ID8-L4 cells were selected for use in an *in vivo* model.

**Fig. 2:**
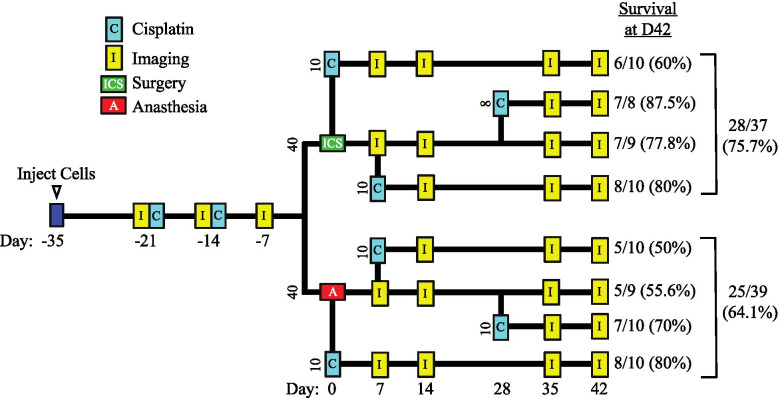
Schematic summary of the overall treatment and imaging schedule for the *in vivo* study. All mice were innoculated with ID8-L4 cells (Day -35) and were imaged (I) and treated with cisplatin (C) 2 and 3 weeks (Days -21 and -14) later. The animals were imaged 4 weeks after innoculation (Day -7) and one week later (Day 0) were divided into 2 primary treatment arms; one arm (*n* = 40 mice) underwent interval cytoreductive surgery (ICS) and the other arm (*n* = 40) were anesthetized (A) but left intact. At Day 0, a subset of 10 mice from each arm was treated with cisplatin. One week later (Day 7), all mice were imaged and a second subset of 10 animals from each arm was treated with cisplatin. One week later (Day 14) all mice were reimaged. Two weeks later (Day 28), a third subset of 8 or 10 mice from the remaining animals in each arm were treated with cisplatin and the remaining animals (*n* = 9) were left untreated. All groups were reimaged 35 and 42 days after surgery/anesthesia. The number and percentage of mice surviving to day 42 in each group and the two primary arms (ICS and A) is indicated

Immunocompetent mice were inoculated with a total of 2x10^6^ ID8-L4 cells, with 1x10^6^ cells injected into the right ovarian bursa and 1x10^6^ cells dispersed into the abdominal cavity. All mice received the first of 2 weekly injections of cisplatin 14 days after inoculation to simulate NACT exposure (see Fig. 2 for treatment schematic). As determined in a preliminary study, 2 weekly courses of cisplatin were found to reduce tumor mass as effectively as 3 courses; therefore, we elected to use 2 pre-surgery cisplatin courses in this study. Two weeks after the second cisplatin treatment, mice were stratified into eight groups based upon bioluminescence imaging performed 7 days earlier, which considered both evidence of seeding beyond the orthotopic tumor and overall luciferase expression levels. At this time, four groups underwent ICS consisting of removal of the right ovarian bursa tumor mass and the other four groups were anesthetized for an equivalent duration but left intact. Within each of these two primary arms, animals were assigned to receive cisplatin at post-operative day 0, or to delay this treatment to day 7 or 28 as outlined in Fig. 2. Control animals in each arm received no peri- or post-surgical chemotherapy. Tumor progression was monitored through live bioluminescence imaging performed until day 42 following surgery/anesthesia.

A total of 40 mice underwent ICS and 40 mice were anesthetized with no surgery. These were further subdivided to form the 8 treatment groups. While an *n*=10 per group was targeted, this number was reduced by death or euthanasia due to disease progression. A slightly greater percentage of animals receiving ICS survived to study endpoint on day 42 as compared to animals receiving anesthesia (75.7 vs. 64.1% respectively) but this was not statistically significant (*p* = 0.32; Fig. 2). Postsurgical cisplatin treatment did not improve survival to day 42 in either the ICS arm (*p* = 1.0) or anesthesia control arm (*p* = 0.70).

**Fig. 3:**
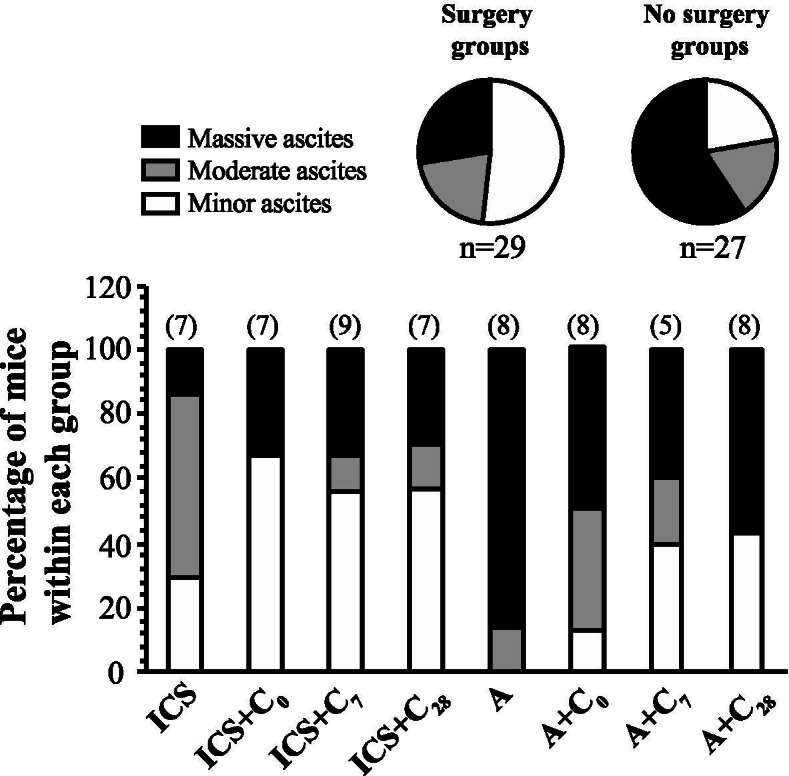
Impact of ICS and cisplatin on ascites accumulation. Ascites volume was measured at the time of necropsy on day 42 or at required euthanasia because of complications of disease progression. Volumes were categorized as minimal (≤1.0 ml), moderate (> 1.0 ml and <10 ml), or massive (≥10 ml). Pie charts show the relative proportion of mice with each category of ascites volume in the ICS (surgery) arm and anesthesia control (A) arm (χ^2^_(2, n=56)_=6.552, *p* = 0.0378). The stacked bar graphs present the relative distribution of ascites categories within each individual treatment group. The numbers in parentheses at the top of each bar indicate the number of animals measured per group. C_0_ = cisplatin on day 0, C_7_ = cisplatin on day 7, C_28_ = cisplatin on day 28

The ID8 cancer model recapitulates many aspects of high-grade serous ovarian adenocarcinoma, including the accumulation of ascitic fluid [24]. Ascites volume was measured at the time of necropsy (day 42) or euthanasia, and categorized as minor (1 ml or less), moderate (greater than 1 ml but less than 10 ml) or massive (10 ml or greater). Mice that did not receive ICS had a greater incidence of massive ascites volume than those receiving surgery (*p* = 0.039), with the greatest percentage of mice developing massive ascites found in animals that did not receive ICS or further cisplatin treatment (Fig. 3).

**Fig. 4:**
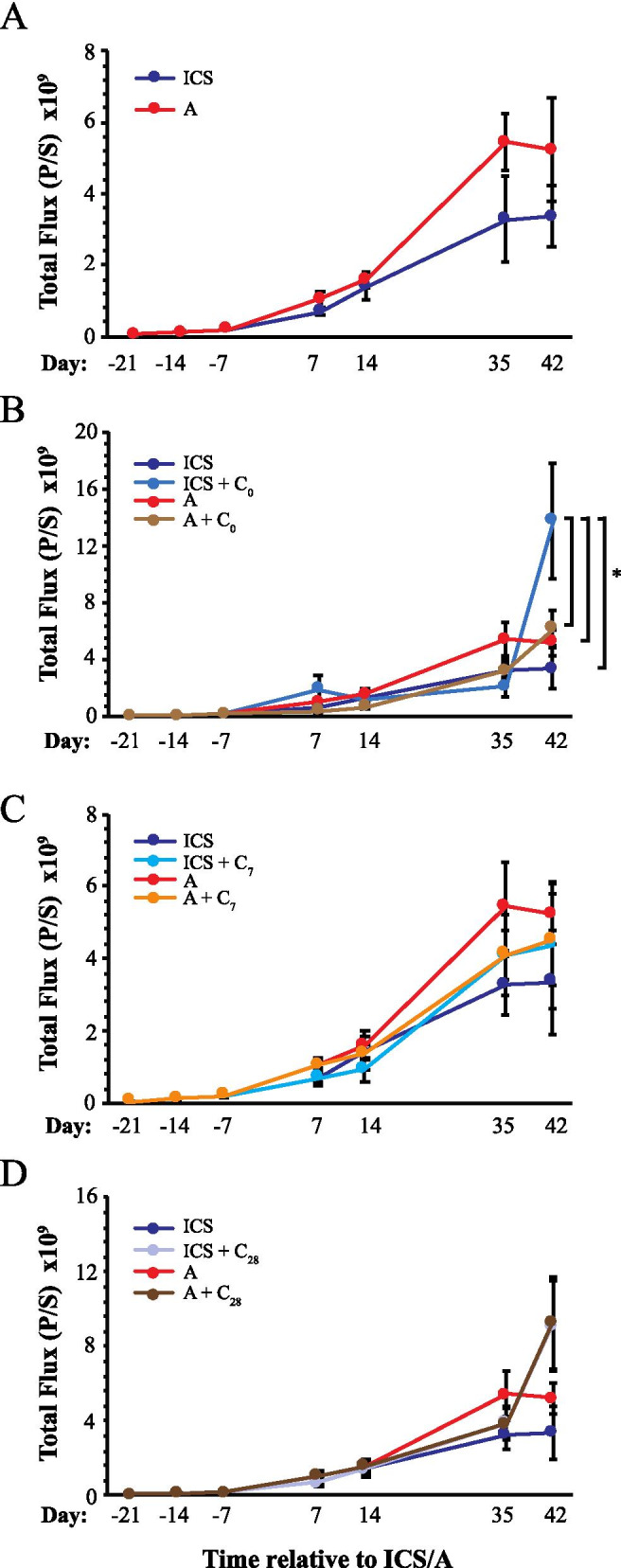
Impact of ICS on tumor burden as determined by bioluminescence measurements across all imaging sessions. Results are presented to compare the impact of ICS vs. no surgery alone (**A**) or combined with cisplatin administered on day 0 (**B**), 7 (**C**), and 28 (**D**). Points indicate the mean ± SEM. Data collected at each imaging time point were analyzed by ANOVA follow by Fisher LSD test, *p<0.05. Abbreviations are defined in the legend to Fig. 3

**Fig. 5:**
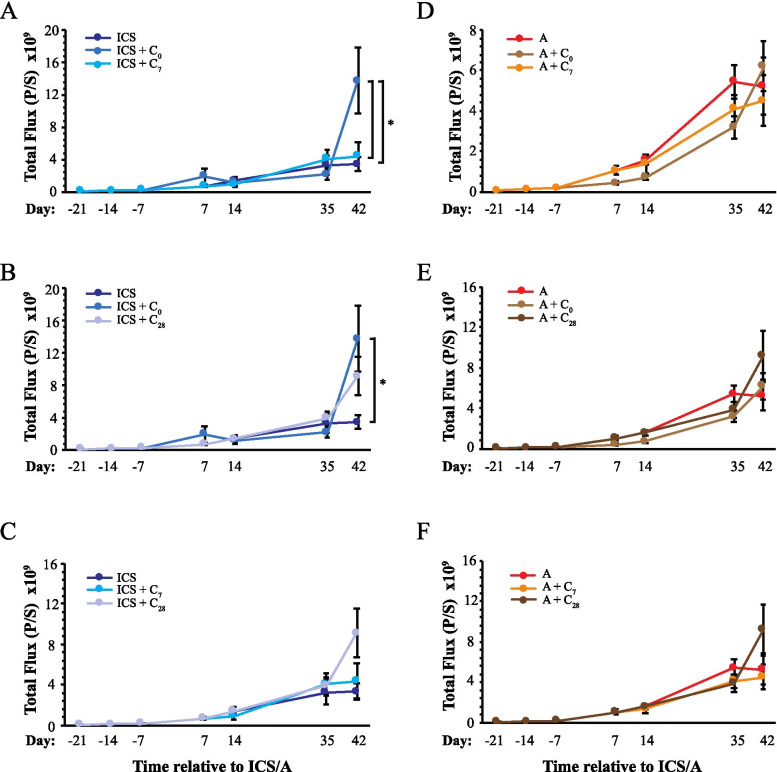
Impact of peri- and post-surgical cisplatin treatment on tumor burden. Bioluminescence data are shown to compare the impact of day of cisplatin administration within groups that had received ICS (**A-C**) or no surgery (**D-F**). **A** and **D** Comparison of Day 0 vs. Day 7 cisplatin administration. **B** and **E** Comparison of Day 0 vs. Day 28 cisplatin administration. **C** and **F** Comparison of Day 7 vs. Day 28 cisplatin administration. Points indicate the mean ± SEM. Data collected at each imaging time point were analyzed by ANOVA followed by Fisher LSD test, *p<0.05. Abbreviations are defined in the legend to Fig. 3

**Fig. 6:**
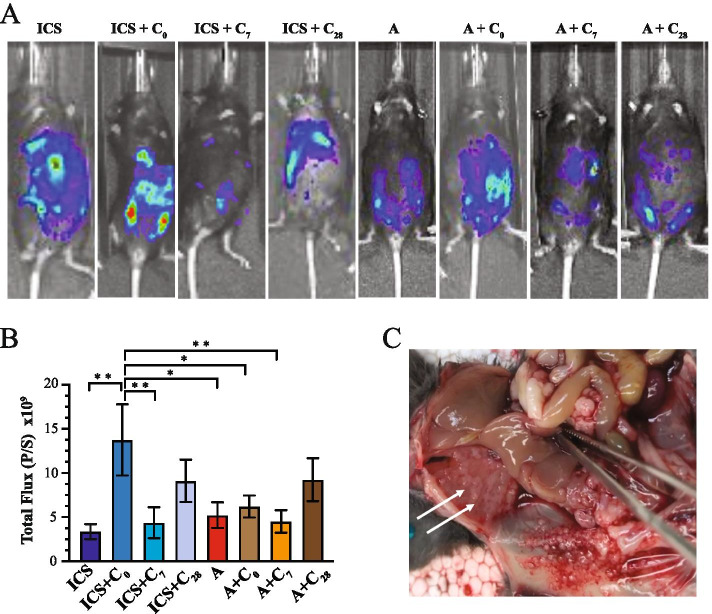
Summary of Day 42 imaging results and representative images. **A** Representative imaging of tumor burden measured on day 42. **B** Bar chart showing results of imaging acquired on day 42. Bars represent the mean ± SEM. Data collected at each imaging time point were analyzed by ANOVA follow by Fisher LSD test; *p<0.02, **p<0.01. Abbreviations are defined in the legend to Fig. 3. **C** Image showing extensive peritoneal dissemination of tumor seeding within the abdominal cavity. Arrows show deposits along the posterior diaphragm surface

Results of bioluminescence imaging of tumor burden are summarized in Figs. 4, 5 and 6. ICS without further cisplatin treatment appeared to decrease tumor burden measured on day 35 and 42 compared to anesthesia-only controls, but this was not statistically significant (Fig. 4A). Post-surgery day administration of cisplatin had no apparent impact on tumor burden in either the surgery or anesthesia arms (Figs. 4 and 5) and no differences were detected between day 7 or day 28 administration of cisplatin (Fig. 5). Statistically significant differences in tumor burden across treatment groups were found only at day 42 (Fig. 6). Remarkably, the highest level of tumor burden was found in mice that had received cisplatin at the time of ICS (ICS = C_0_; Figure 6). This level was statistically higher than those measured in mice that had received ICS with no further cisplatin treatment or who had received cisplatin on day 7 (ICS + C_7_). Additionally, cisplatin at the time of ICS resulted in higher bioluminescence levels than that measured in anesthesia control mice that had received cisplatin at the same time (A + C_0_), 7 days later (A + C_7_), or that had not been treated with further cisplatin (A). Upon necropsy, animals with advanced disease and high luciferase activity exhibited extensive peritoneal distribution of seeding, particularly along the diaphragmatic surfaces that is characteristic of advanced ovarian cancer (Fig. 6C).

## Discussion

Primary cytoreductive surgery for advanced stage ovarian cancer harbors a survival adavantage if tumor burden can be decreased to optimal status, defined as nodules smaller than 1.0 cm^3^ [3]. Complete cytoreduction with elimination of all macroscopic disease adds further survival benefit over optimal cytoreduction. In patients with apparent unresectable disease at presentation or who have significant baseline morbidities, NACT can be administered followed by interval cytoreductive surgery, and additional post-operative consolidation chemotherapy [3].

Unfortunately, the majority of patients with advanced ovarian malignancies will experience a recurrence and ultimately tumors that initially were platinum sensitive, will become platinum resistant. It remains controversial as to whether NACT augments the emergence of chemoresistant tumor cells by selectively eliminating only chemosensitive tumor cells, allowing a greater proportion of chemoresistant cells available to expand [29]. Furthermore, while clinical studies indicate that a shorter interval to resumption of chemotherapy may improve survival [30–34], it is less clear whether heated intraperitoneal chemotherapy (HIPEC) at the time of surgery is beneficial. Furthermore, perturbation of the immune system resulting from surgical wounding may impact residual disease progression. Recent studies have shown that altered myloid cell differentiation resulting in an immunosuppressive state can result from surgery [35] or myocardial infarction [36] to promote the outgrowth of breast cancer cells.

In this study, we developed a mouse model of NACT using syngeneic murine ID8 ovarian cancer cells. In an orthotopic model, these cells were shown to create primary tumors morphologically similar to papillary serous high-grade carcinoma and to produce diffuse peritoneal seeding and ascites production similar to the clinical presentation of high-grade serous ovarian cancer [24]. Given the increasing use of a combination of NACT and interval surgery, the development of reliable animal models will be important to further understanding of the impact NACT has on tumor biology.

Our group previously examined the role for earlier administration of adjuvant chemotherapy using a murine ovarian cancer primary surgery model [17]. In that study, we found that surgery accelerated residual tumor growth and that this effect was minimized through the administration of earlier cisplatin on post-operative day 7 [37]; however, in this model, surgery consisted of wounding without extirpation of tumor cells. In this model, we also found that surgical wounding impaired the efficacy of intraperitoneal cisplatin treatment administered on the day of wounding. In NACT, cells are exposed to platinum based-chemotherapy for several cycles and thus the impact of surgery and timing of cisplatin may differ from that of chemo-naïve cells in primary surgery.

When compared to animals that had not received ICS and tumor cytoreduction, ICS did not appear to reduce tumor burden appreciably, although it reduced the number of animals that developed massive ascites, as did peri- or post-surgical cisplatin treatment. Surprisingly, intraperitoneal cisplatin treatment on the day of ICS increased tumor burden measured 6 weeks later. This may be attributable to the surgical procedure as the tumor burden was significantly greater than that measured in animals treated similarly but that had not undergone ICS. Moreover, the increase in tumor burden was prevented when delaying cisplatin treatment by 7 days. Importantly, surgical wounding induces a cascade of cytokines and growth factors, many of which overlap with cellular processes related to cancer cell growth and metastasis, including angiogenesis [38]. Our observation of improved control of ascites in animals who undergo wounding compared to control animals suggests there may be a relationship to those growth factors recruited for wound healing and the membrane stabilization of neovascularization within residual tumor deposits following sub-optimal cytoreduction in our model. The burden of ascites was least in those animals who received cisplatin on day 0 suggesting a possible synergistic effect of platinum and surgical wounding that is not fully understood. It may be that those factors related to vascular permeability are blocked while permitting the recruitment of pathways related endothelial stabilization. In a trial of adding bevacizumab to chemotherapy and continuing it as maintenance therapy, women who have stage IV disease and sub-optimal cytoreduction experience the greatest benefit from the addition of bevacizumab, a mono-clonal antibody targeting vascular endothelial growth factor which regulates angiogenesis [39]. It is feasible that the combination of interval surgical wounding and peri-operative cisplatin in this model of sub-optimal cytoreduction affects antiangiogenic pathways contributing to improved control of ascites. Further work is needed to explore this relationship of angiogenic pathways at interval surgery in those who have received extensive chemotherapy pre-operatively.

Our findings are most intriguing when contrasted with our previous work in surgical wounding of animals in a model of primary surgery for advanced ovarian cancer [17]. In animals naïve to cisplatin, surgical wounding resulted in accelerated tumor growth whereas in the current study there was no significant effect of wounding on final tumor burden. There are several proposed mechanisms that could explain the difference. Firstly, it is known that NACT followed by ICS increases platinum-resistant disease [40] or decreases treatment-free interval [41]. Chemo-resistant clones may have be less sensitive to the cascade of growth factors at the time of surgical wounding compared to those who are not exposed to pre-operative chemotherapy due to changes in gene expression after treatment. Additionally, animals exposed to pre-operative cisplatin likely have impaired immune system functioning due to the hematologic toxicity of platinum derivatives. The systemic inflammatory response to surgical wounding and growth of metastatic disease has been linked to immune driven processes in animal models of other solid tumors [35]. We hypothesize that pre-operative chemotherapy may alter this pathway of accelerated tumor growth as a consequence of immunosuppressive effects from cisplatin.

We expected to observe that excision of the primary ovarian mass would lead to accelerated growth compared to animals that did not undergo surgery but this was not observed. Large primary tumors secrete factors like angiostatin, endostatin and thrombospondin [42] that inhibit growth of distant disease and removing the dominant mass has been shown to remove this inhibition and lead to increase proliferation of distant disease [43]. Animal models of breast cancer have shown that primary surgery leads to altered expression of genes related to tumor adhesions, invasion and angiogenesis [44]. It will be important to evaluate circulating levels of these factors in clinical specimens of women undergoing surgery for advanced ovarian cancer. Our findings suggest there may be important differences in the systemic response and immune microenvironment of women undergoing primary versus interval cytoreduction and they should be explored to identify potential therapeutic targets that can be exploited to maximize outcomes.

There are several limitations to the current study. The results of animal models are inherently limited in their translation to human outcomes, particularly in the setting of timing of chemotherapy and mimicking clinical conditions. Much work has been done to determine dose equivalences across various species; however, there has been little to no research into timing of chemotherapy in mice compared to humans. Our choice of using post-operative day 28 cisplatin as the standard of care control is based on the clinical condition of resuming adjuvant treatment approximately 4 weeks post-operative; however, these pharmacokinetics do not necessarily translate directly to mice. Unlike in the clinical setting, we only administered a single post-operative dose of cisplatin which may have limited our results. Our primary outcome was burden of disease using bioluminescence as a surrogate which is not in keeping with most clinical studies which assess survival, which is not ethically feasible to assess in animal studies. High burden and distribution of intraperitoneal disease is likely related to survival in human patients and, therefore, we believe bioluminescence is an appropriate outcome for the purposes of this research question. Finally, the contemporary definitions of ‘optimal’ cytoreduction and residual disease have not been quantified in animal models and therefore it is hard to make assumptions regarding the residual disease following resection of the primary tumor in this model. It is not feasible to subject mice to an extensive cytoreductive procedure in this setting and as such we assume that there is macroscopic disease remaining following unilateral oophorectomy and our findings are within the context of ‘sub-optimal’ cytoreduction.

Despite its limitations, this animal model replicates the conditions of advanced high-grade serous ovarian cancer as best as could be achieved in an animal setting and this is one of the first studies to describe animal models of NACT/ICS in ovarian cancer. Our cell lines were sensitive to platinum-based chemotherapy, animals developed diffusely metastatic disease prior to initiating NACT, many developed ascites at recurrence and findings at necropsy mimic the volume and distribution of disease seen in peritoneal malignancies. Additional strengths include the contrast in results to our study in primary surgical wounding, suggesting that there are mechanisms of wound healing and tumor biology unique to animals pre-treated with cisplatin. The present study was designed to determine the impact of interval cytoreductive surgery and timing of cisplatin on disease progression and precluded our ability to study molecular changes in the tumor at early critical time points. Future mechanistic studies based upon our findings are needed to identify key inflammatory signalling networks involved. Such studies may identify targets that can be blocked in order to prevent potential disease-promoting processes triggered at primary surgery.

In addition, our findings with this model suggest that cisplatin administered 7 days after surgery resulted in better disease control compared to administration at 28 days. Clinical studies correlating survival with elapsed time from interval cytoreductive surgery and resumption of chemotherapy are lacking. Such retrospective clinical studies can be problematic as multiple variations in subsequent treatments and other factors can influence outcome.

## Conclusions

In summary, we have developed a mouse model of advanced ovarian cancer mimicking NACT and interval surgical cytoreduction. Surgical wounding did not result in accelerated growth of metastatic disease but did impair the effects of peri-operative cisplatin. There appears to be a relationship between surgical wounding and control of ascites and this effect is most pronounced in combination with cisplatin at the time of wounding. Further work is needed to explore these findings in clinical specimens as to maximize surgical outcomes for women undergoing cytoreductive procedures for advanced ovarian malignancies.

## Materials and methods

### Cell culture

Luciferase-expressing ID8 cells were previously generated [17] and verified to be mycoplasma free using a PCR-based mycoplasma detection kit (Applied Biological Materials Inc., Richmond, BC, Canada). Parental and luciferase-expressing cells were maintained in RPMI 1640 medium supplemented with 5% heat inactivated fetal bovine serum, 100 units/mL penicillin and 100 μg/ml streptomycin (all from Invitrogen, Burlington, ON, Canada). Medium for ID8-luciferase expressing cells was also supplemented with 1 μg/ml puromycin (Invitrogen) to maintain selection of transfected cells. Cells were maintained at 37°C in a humidified incubator with 5% CO_2_. For inoculation into mice, cells were harvested, counted using a TC20 automated cell counter (BioRad, Mississauga, ON, Canada) and resuspended in phosphate-buffered saline (PBS) to a concentration of 200,000 cells/μl.

### XTT dye reduction assay

Cells were seeded in 96-well plates at a density of 2x10^3^ cells per well. After 24 h, cisplatin (Sigma, St. Louis, MO, USA) was added to a final concentration of 0, 10, 25 or 50 μM and cell number was assessed 24, 48, and 72 h later by XTT dye-reduction assay. Briefly, 50 μl XTT solution (1 mg/ml, Invitrogen) were added to each well. Following a 3h incubation in the tissue culture incubator, levels of reduced XTT reflecting the number of viable cells, were measured at 492 nM absorbance using a microtiter plate reader (Infinite M200; Tecan Life Sciences, Männedorf, Switzerland).

### Animals

Female C57BL/6J mice (8-10 weeks old) were obtained from Charles River Laboratories (Sherbrooke, QC, Canada) and group-housed under standard conditions in compliance with Canadian Council on Animal Care guidelines. All animals were maintained in a 12-12 h light-dark schedule with food and water provided *ad libitum*. All animal procedures were approved by the University of Toronto Animal Care and Use Committee. Animals were acclimated to the housing conditions for 7-10 days before initiation of experimental procedures.

### *In vivo* ID8 cell innoculation

Cell inoculation was performed under 1.5-2.5% isofurane anesthesia (Baxter, Deerfield, IL, USA) using sterile technique to recapitulate the clinical scenario of advanced disease with a primary tumor and carcinomatosis. Briefly, a mid-dorsal incision (1.0 cm) was made through the skin and reflected to reveal the musculature slightly right of midline and overlying the right ovary. A small incision through the musculature was made and the ovarian bursa was externalized for intrabursal injection of 5 μl PBS containing 1x10^6^ cells. The bursa was returned to the abdominal cavity and an additional 5 μl of the cell suspension was dispersed into the peritoneal cavity through the incision. Both the abdominal musculature and skin were closed with absorbable sutures with the skin sutures additionally bonded with Ethicon Dermabond topical adhesive to reduce the risk of wound dehiscence. At the time of surgery, animals received 1 mg/kg body weight buprenorphine (0.3 mg/ml) and 2 mg/kg body weight meloxicam (0.2 mg/ml) analgesia. Buprenorphine dosing was repeated daily for 2 days following surgery, whereas meloxicam dosing was repeated only on the day following surgery. Cisplatin was dissolved in PBS filter-sterilized, and injected intraperitoneally at a dose of 2 mg cisplatin/kg body weight at the times specified. This dose corresponds to a clinical dose between 75-100 mg/m^2^ [45].

### Interval cytoreductive surgery (ICS)

ICS consisted of extirpation of the tumor-containing right uterine horn adnexa under 1.5-2.5% isoflurane anesthesia. The surgical procedure was similar to the procedure described for cell inoculation. Briefly, a mid-dorsal incision (1.0 cm) was made at the previous site and reflected to enable an incision in the musculature overlying the right ovary. The ovarian bursa tumor was externalized and ligated before excision. The uterine horn wound was cauterized and returned to the abdominal cavity. Both the abdominal musculature and skin were closed with absorbable sutures with the skin sutures additionally bonded with Ethicon Dermabond topical adhesive. Animals received daily buprenorphine analgesia on the day of surgery until postoperative day 2. Control mice were subjected to isoflurane for an equivalent duration to that of mice undergoing ICS and were administered the same analgesia regimen. Mice were monitored regularly for tumor growth, ascites accumulation and postoperative complications. Animals were euthanized by CO_2_ inhalation at post-operative day 42 (D 42) from either interval surgery or anesthesia alone, or if they exhibited signs of distress, poor health, greater than 20% weight loss or excessive ascites prohibiting full mobility. Ascites volume was measured, and for those animals randomized to anesthesia without ICS, the right adnexal tumor was excised and weight was recorded.

### Bioluminescence imaging

Two ID8 luciferase expressing clonal cell sublines, ID8-L4 and ID8-L11, were subjected to bioluminescence imaging. Briefly, cells were seeded in 100 μl medium into a 96-well plate and just prior to imaging using an IVIS Spectrum In Vivo Imaging System (Perkin Elmer, Rodgau, Germany), an equal volume of *in vivo* glow solution (D-Luciferin, Promega) was added to each well. Images were obtained with a 12.8 cm field of view, 2x2 binning factor and an exposure time of 1 second. As recommended by the manufacturer, the auto-exposure setting was used to automatically set the exposure time, f/stop and binning to keep the signal within an optimal range for quantification and to avoid overexposure during image acquisition. Auto-exposure sensitivity settings used for the snapshot image were adjusted to obtain a minimal target count of 3000. Luminescence was measured as total flux (photons per second (P/S)).

*In vivo* live bioluminescence imaging of tumor burden was performed using an IVIS Spectrum *In Vivo* Imaging System. Animals were anesthetised with 1.5-2.5% isoflurane inhalation and given an intraperitoneal injection of 165 mg/kg body weight D-luciferin (Promega; 30 mg/ml sterile saline) 3 min immediately prior to imaging. Mice were imaged in batches of 4. Images were obtained with a 12.8 cm field of view, 4x4 binning factor and an exposure time ranging from 0.5-1 second.

### Statistical analysis

Unless otherwise stated, data are presented as mean ± SEM. Statistical analyses were performed using Prism for MacOS, version 8.4.3 (GraphPad Software). Categorical data were analzed by Chi square or Fishers Exact Probability Test. Continuous data were analyzed by ANOVA followed by Fisher’s LSD test. *In vivo* bioluminescence data were analyzed within each imaging timepoint. Statistical significance was defined as *p*<0.05. Research data are not shared.

## Data Availability

The data used and analysed during this study are available from the corresponding on a reasonable request.
